# Uniconazole-mediated growth regulation in *Ophiopogon japonicus*: yield maximization vs. medicinal quality trade-offs

**DOI:** 10.3389/fpls.2025.1542539

**Published:** 2025-07-15

**Authors:** Xiaoyang Cai, Wenjing Li, Heling Fan, Jiaming Zhang, Haohan Wang, Yan Qing, Min Li, Yan Gou

**Affiliations:** ^1^ School of Pharmacy, Chengdu University of Traditional Chinese Medicine, Chengdu, China; ^2^ Sichuan Research Center for Demonstration Project of Entire Industrial Chain of Genuine Medicinal Materials, Chengdu, China; ^3^ Sichuan Institute for Drug Control/NMPA Key Laboratory for Quality Evaluation of Traditional Chinese Medicine (Traditional Chinese Patent Medicine), Chengdu, China

**Keywords:** *Ophiopogon japonicus*, uniconazole, medicinal quality, plant hormones, plant growth regulators (PGRs)

## Abstract

**Introduction:**

*Ophiopogon japonicus* (L. f.) Ker-Gawl., commonly known for its tuberous roots, is a renowned medicinal plant widely used in traditional medical systems across China, Japan, and parts of Southeast Asia. In China in particular, Ophiopogonis Radix has been employed for thousands of years as both a herbal remedy and a health-promoting food, embodying a long-standing tradition of dual medicinal and dietary use. Based on geographic origin, it is typically classified into two main types: “Chuanmaidong” (CMD) from Sichuan and “Zhemaidong” (ZMD) from Zhejiang. This study investigates the impact of foliar-applied Uniconazole, a triazole-based plant growth regulator, on the agronomic traits and medicinal quality of *Ophiopogon japonicus* (cv. Chuanmaidong No.1) under field conditions in Sichuan, China. The research addresses a critical question in medicinal plant cultivation: can yield enhancement via plant growth regulators be achieved without compromising pharmacological quality?

**Methods:**

Uniconazole was applied at rates ranging from 7.5, 15, and 30 kg/hm². Plant morphological traits, tuber yield components, bioactive compound contents, and environmental residues were systematically evaluated. Hormonal profiling and degradation kinetics were also assessed to elucidate physiological mechanisms and ecological safety.

**Results:**

Uniconazole application inhibited vegetative growth, reducing plant height and leaf biomass. However, it markedly increased tuber yield—by up to 101.59%—through hormone-mediated morphological remodeling. This was driven by disruptions in endogenous hormone homeostasis, particularly in Abscisic Acid (ABA) - Gibberellic acid 3 (GA_3_) balance and Indole-3-Acetic Acid (IAA) - Zeatin Riboside (ZR) coordination, promoting the transformation of root shapes from standard spindle forms to cylindrical or dumbbell types. Dimensional traits improved significantly: root diameter increased by 12.36%, length by 21.75%, and single tuber dry weight by 49.53%. Despite modest increases in polysaccharide and flavonoid levels, total saponins and ophiopogonin D—key pharmacologically active compounds—declined by 35.90% and 63.94%, respectively. Environmental residue analysis showed first-order degradation kinetics, with half-lives of approximately 19.7 days in both soil and root tissue, and final residues falling below detection thresholds.

**Conclusion:**

While Uniconazole enhances short-term economic returns through yield amplification, it poses substantial challenges to medicinal quality and regulatory compliance. The induced morphological deviations complicate adherence to Chinese Pharmacopoeia identification standards and may increase adulteration risks. Most concerning is the sharp reduction in saponins, which undermines clinical efficacy and pharmaceutical processing. This study calls for urgent policy reforms, including mandatory quantification of bioactive markers and routine residue monitoring, to safeguard the integrity of medicinal plant supply chains. A balanced cultivation paradigm is essential—one that reconciles agricultural intensification with the core therapeutic values of medicinal crops: efficacy, safety, and authenticity.

## 1 Introduction

With the increasing global awareness of health and the shift in medical paradigms, Ophiopogonis Radix (the dried product derived from the root tuber of *Ophiopogon japonicus* (L. f.) Ker Gawl.) has gained widespread recognition in the global market as an important natural medicine due to its specific therapeutic effects and safety (Jin et al., 2024). It is known for its pharmacological properties, including lowering blood sugar (Ding et al., 2012; Wang, 2013), protecting the cardiovascular system (Zheng et al., 2009; Lan et al., 2013), enhancing immunity (Li Xiong et al., 2011), anti-aging effects on the skin (Li Xiong et al., 2011), anti-inflammatory properties (Song et al., 2010), and anti-tumor effects (Zhang et al., 2012; Chen et al., 2013). Due to its efficacy and safety (Jin et al., 2024), Ophiopogonis Radix is widely used in at least 25 countries and regions, including China, Japan, Germany, Vietnam, India, the United States, Malaysia, and South Korea. According to data from the General Administration of Customs of China, from 2018 to 2022, the total export of Ophiopogonis Radix exceeded 6,000 tons. Santai County in Sichuan Province is a key production area for Ophiopogonis Radix, with an annual output reaching up to 15,000 tons, accounting for over 90% of the domestic market. Advances in new varieties, cultivation techniques, and nutrient management (Hai et al., 2024) have led to increased yields, particularly through the application of plant growth regulators (Zhang et al., 2021), which can significantly enhance the yield of *Ophiopogon japonicus*. Therefore, it is essential to move beyond the traditional linear perspective of “enhancing both quality and yield.” The use of plant growth regulators like uniconazole may present a double-edged sword for medicinal crops: can they increase yield without compromising key therapeutic compounds? Moreover, does the interaction between the degradation rate of uniconazole within the plant-soil system and the harvest interval effectively ensure that its residue levels in both the medicinal product and the environment remain below safety thresholds?

Plant growth regulators (PGRs) are generally recognized for their ability to promote metabolism (carbon and nitrogen), delay or accelerate plant aging and abscission, and increase crop yields. Uniconazole [(E)-(RS)-1-(4-chlorophenyl)-4, 4-dimethyl-2-(1H-1, 2, 4-triazol-1-yl) pent-1-en-3-ol] is a highly active, low-toxicity, and low-residue plant growth regulator that follows the introduction of paclobutrazol (Fletcher and Hofstra, 1990; Fletcher et al., 2010). Compared to paclobutrazol, Uniconazole has higher activity, a shorter residual period, and does not adversely affect subsequent crops (Bekheta et al., 2012; Wan Yan et al., 2012). Studies have shown that triazole compounds can influence the isoprenoid pathway by inhibiting gibberellin (GA) synthesis while increasing levels of abscisic acid (ABA) and cytokinins (CK) (Soumya et al., 2017). This mechanism helps protect plants from various stresses (Fletcher et al., 2010), particularly by enhancing resistance to abiotic stresses such as flooding (Qiu et al., 2005; Wang et al., 2022b), drought (Keshavarz and Khodabin, 2019; Jiang et al., 2021), high temperatures (Zhou and Leul, 1999), low temperatures (Hu et al., 2022), and salinity (Al-Rumaih and Al-Rumaih, 2007; Du et al., 2024). Uniconazole has been widely applied in crops such as corn (Ahmad et al., 2021), wheat (Ahmad et al., 2019), soybeans (Zhou et al., 2021), and rapeseed (Zuo et al., 2020), resulting in increased yields. Furthermore, some studies indicate that triazole compounds can promote starch accumulation by increasing CK and ABA levels while reducing GA and IAA levels (Rahim et al., 2011; Liu et al., 2015). However, the potential effects of Uniconazole application on the accumulation of quality indicators, such as polysaccharides in *Ophiopogon japonicus*, remain to be further investigated. Additionally, the impact of Uniconazole application on endogenous hormones in different parts of *Ophiopogon japonicus* is still unclear.

Plant growth regulators (PGRs) play a critical role in enhancing plant growth, yield, and quality. These compounds influence key metabolic pathways, improving plant development and productivity. For example, in crops such as pepper (Chaudhary et al., 2006), strawberry (Rathod et al., 2021), and watermelon (Kumar et al., 2022), PGRs have been shown to improve plant architecture, boost yield, and enhance nutritional attributes such as acidity, ascorbic acid content, and total sugar levels. However, their widespread application in medicinal plant cultivation has raised safety concerns (Chung et al., 2022; Zhang et al., 2022a). Uniconazole, a commonly used PGR, has demonstrated significant benefits in increasing the yield of medicinal crops. Yet, the potential risks associated with overuse—such as yield reduction, quality deterioration, and excessive residue—must not be overlooked (Xu et al., 2018; Zhou et al., 2022; Chauhan et al., 2024). This study focuses on the scientific application of uniconazole in the cultivation of *Ophiopogon japonicus*, a medicinal plant. Given its inherent toxicity and persistence, indiscriminate use in current farming practices poses risks, including poor quality and residue levels that may exceed safety thresholds. Moreover, the underlying mechanisms of its action and its behavior in the soil–plant system remain insufficiently understood. This research aims to elucidate the combined effects of uniconazole treatment on plant morphology, endogenous hormone dynamics, accumulation of active compounds, and residue patterns in the soil–plant continuum. It particularly explores the interconnected “dose–response–residue” relationship and its impact on balancing yield and medicinal quality. By clarifying these links, the study provides essential insights for the safe and efficient use of PGRs in medicinal plant production. The results are expected to fill critical knowledge gaps regarding the formation of therapeutic compounds and the dissipation of chemical residues. This work offers a theoretical basis for developing precision application strategies that simultaneously optimize plant structure, metabolic regulation, and environmental safety. In doing so, it supports a shift from traditional, experience-based cultivation to a modern, science-based paradigm rooted in hormone signaling, secondary metabolite regulation, and ecological risk assessment. The proposed agronomic strategy—emphasizing reduced input, residue control, enhanced quality, and improved efficiency—will have practical value in standardizing PGR use and ensuring the quality and safety of traditional Chinese medicinal products.

## 2 Materials and methods

### 2.1 Experimental design

This study was conducted at the *Ophiopogon japonicus* research demonstration base in Santai County, Sichuan Province (longitude 104°57’44”, latitude 31°24’35”). The region has a subtropical monsoon climate, with an average annual temperature of approximately 17.8°C, total annual sunshine hours reaching 1,490, an average annual precipitation of about 938.4 mm, and an average relative humidity of 79% (The relevant data were obtained through the Sichuan Meteorological Service http://sc.cma.gov.cn/). The soil is sandy loam, characterized by a pH of 7.05, organic matter content of 10.3 g/kg, ammonium nitrogen content of 28.2 mg/kg, available phosphorus content of 81.2 mg/kg, and available potassium content of 124.8 mg/kg. Uniconazole had not been applied to the soil during previous cultivation at the experimental site, ensuring that the soil background would not interfere with the current experimental treatments.

The objective of this research is to assess the effects of different doses of Uniconazole on the growth, yield, medicinal quality indicators, and hormonal changes in various parts of *Ophiopogon japonicus*, as well as the residual levels of Uniconazole in Ophiopogonis Radix and soil. All treatments employed uniform fertilizer management, using 1.5 t/hm² of commercial organic fertilizer (N + P_2_O_5_ + K_2_O > 5%, organic matter > 45%, produced by Mianyang Keya Agricultural Development Co., Ltd.) and 0.6 t/hm² of compound fertilizer (N: P_2_O_5_: K_2_O = 17:17:17, total nutrients > 51%, produced by Guizhou Xiyang Industrial Co., Ltd.).

The fertilization experiment followed a completely randomized block design, consisting of four treatment groups. Each treatment was replicated three times, totaling 12 plots, with each plot measuring 12 square meters. The uniconazole application rates are shown in **Table 1**. For each treatment, uniconazole was diluted in 1,200 L of water prior to application. The selected concentrations were based on existing usage levels in the production region as well as the recommended application rates provided by the uniconazole product manufacturer. *Ophiopogon japonicus* was planted at a density of 1.5 million plants per hectare using the variety “Chuanmaidong No. 1.” This improved cultivar is known for its tolerance to abiotic stress (Airong et al., 2020; Cheng et al., 2024, 2025), with continued growth during winter low temperatures and broad adaptability to various soil conditions, making it suitable for both medicinal and ornamental purposes. Seeding was conducted on April 13, 2023. Uniconazole was applied on October 20, 2023, coinciding with the onset of root tuber expansion, which was designated as day 0. Standard field management practices were followed throughout the growing season (China Association of Chinese Medicine, 2020), and harvesting was scheduled for March 17, 2024.

**Table 1:** Tables should be placed in the main text near to the first time they are cited.

Treatment code	CK	U1	U2	U3
Uniconazole(kg/hm^2^)	0	7.5	15	30

### 2.2 Methods for determination of agronomic indicators

**Figure 1:**
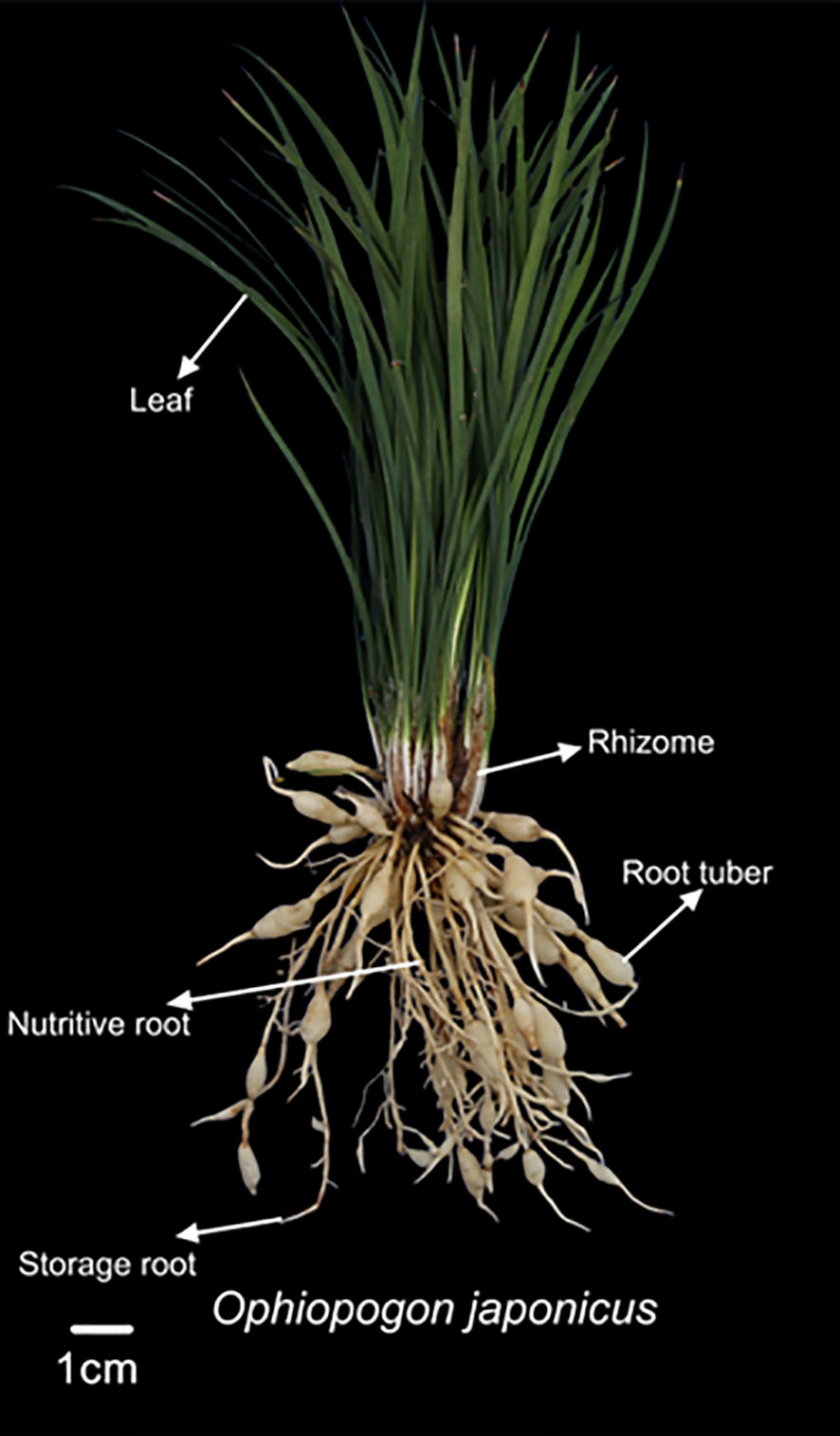
Morphological features of various plant organs in *Ophiopogon japonicus*.

The height of *Ophiopogon japonicus* was measured using a tape measure, and the quantities of nutritive roots, storage roots, and root tubers were recorded. The fresh weights of different parts of the plant (leaves, rhizomes, nutritive roots, storage roots, and root tubers) were measured using a 0.01 g electronic balance, as illustrated in **Figure 1**. Additionally, the length and diameter of Ophiopogonis Radix were determined using a caliper, and the individual weight of each Ophiopogonis Radix was recorded with a 0.01 g electronic balance. Ophiopogonis Radix (OR) refers to the root tubers processed for medicinal use.

### 2.3 Methods for determining quality indicators of ophiopogonis radix medicinal material

#### 2.3.1 Determination of moisture, extractives, total saponins, total flavonoids, and total polysaccharides

The determination of moisture, extractives, and total saponins for Ophiopogonis Radix was conducted according to the methods outlined in the 2020 edition of the Pharmacopoeia of China (Commission C P, 2020). The total flavonoid content in the medicinal material was measured using ultraviolet spectrophotometry, with hesperidin as the reference standard (Lin, 2014). The total polysaccharide content was determined using the sulfuric acid-anthrone colorimetric method (Wang et al., 2016).

#### 2.3.2 Determination of ophiopogonin D, methylophiopogonanone A and methylophiopogonanone B

Standards for Ophiopogonin D (reference substance number Wkg22061609), Methylophiopogonanone A (reference substance number Wkq24021807), and Methylophiopogonanone B (reference substance number Wkq23072006) were purchased from Sichuan Weikegi Biological Technology Co., Ltd. (Chengdu, China). High-performance liquid chromatography (HPLC)-grade acetonitrile was obtained from Sichuan Cologne Chemical Co., Ltd. (Chengdu, China). HPLC was employed to determine the content of Ophiopogonin D, Methylophiopogonanone A, and Methylophiopogonanone B in the samples.

For the extraction of Ophiopogonin D, 3 g of Ophiopogonis Radix powdered material was placed in a round-bottom flask with 50 mL of methanol. The mixture was refluxed for 2 hours, filtered, and the filtrate was concentrated to dryness. Afterward, 10 mL of water was added to dissolve the residue. The solution was then extracted with water-saturated n-butanol, shaking five times with 12 mL each time. The n-butanol extracts were combined and washed twice with 5 mL of ammonium hydroxide solution. The ammonium solution was discarded, and the n-butanol solution evaporated to dryness. Finally, the residue was dissolved in 80% methanol, transferred to a 5 mL volumetric flask, and filtered through a 0.45 μm membrane filter (Wu et al., 2015).

The chromatographic conditions for Ophiopogonin D included using a C18-bonded silica gel column (250 mm length, 46 mm inner diameter, 5 μm particle size). The mobile phase consisted of water (A) and acetonitrile (B) with a gradient elution as follows: 0–5 min, 38%–55% acetonitrile; 5–15 min, 55%–70% acetonitrile; 15–17 min, 38%–55% acetonitrile; 17–20 min, 70%–38% acetonitrile. The flow rate was 1.0 mL/min, the column temperature was set at 25°C, the drift tube temperature at 105°C, and the gas flow rate at 3.0 L/min. The retention time for Ophiopogonin D was 12.3 minutes.

For the extraction of Methylophiopogonanone A and Methylophiopogonanone B, 3.0 g of Ophiopogonis Radix powder was placed in a conical flask with 25 mL of water and allowed to stand for 24 hours. The mixture was then ultrasonicated (500 W, 40 kHz) for 60 minutes, shaken well, and filtered to obtain the filtrate (Wu et al., 2016).

The chromatographic conditions for Methylophiopogonanone A and Methylophiopogonanone B also utilized a C18-bonded silica gel column (250 mm length, 46 mm inner diameter, 5 μm particle size). The mobile phase consisted of water (A) and acetonitrile (B) at 60% acetonitrile, employing isolation. Detection was carried out at 296 nm, with a flow rate of 1.0 mL/min and a column temperature of 25°C. The retention times for Methylophiopogonanone A and Methylophiopogonanone B were 13.5 minutes and 15.3 minutes, respectively.

#### 2.3.3 Determination of gibberellin 3, abscisic acid, indole-3-acetic acid, and zeatin riboside

The concentrations of gibberellin 3 (GA_3_), abscisic acid (ABA), indole-3-acetic acid (IAA), and Zeatin Riboside (ZR) in different parts of *Ophiopogon japonicus* were determined using a double-antibody sandwich enzyme-linked immunosorbent assay (ELISA). The kits were provided by Nanjing Jianchen Bioengineering Institute. To calculate the relative increase rate of different hormones at different time intervals, use the following formula:


$$\text{Relative Increase Rate }(\%)=\frac{\left(LnC_{2}-LnC_{1}\right)}{t_{2}-t_{1}}\times 100$$

Where: t_1_, t_2_ represent the two time points being compared. *C_1_*, *C_2_* are the growth indicators measured at t_1_, t_2_ (Lamont et al., 2023).

#### 2.3.4 Determination of uniconazole residues

The residual amount of uniconazole was quantified using ultra-high-performance liquid chromatography-tandem triple quadrupole mass spectrometry (UHPLC-MS/MS) in multiple reaction monitoring (MRM) mode, employing the external standard method with a matrix standard curve. Ophiopogonis Radix Preparation: We precisely weighed 3.0 g of Ophiopogonis Radix and placed it in a 50 mL polystyrene centrifuge tube. Then, 15 mL of 1% acetic acid solution was added (10 mL glacial acetic acid in 1000 mL water) and mixed thoroughly. After allowing it to stand for over 30 minutes, we added 15 mL of acetonitrile and shook vigorously for 20 minutes. The mixture was then stored at -20°C for more than 30 minutes. Afterward, a QuEChERS extraction packet containing 7.5 g of a mixture of anhydrous magnesium sulfate and sodium acetate (4:1, m/m) was added, and the contents were mixed. Following an additional 20 minutes of vigorous shaking and centrifugation at 9000 rpm for 8 minutes, 8 mL of the supernatant was collected. We then transferred 1.5 mL to a sample vial and purified it using a dispersive solid-phase extraction tube preloaded with QuEChERS purification packets. Soil Preparation: For the soil sample, 3.0 g was accurately weighed and placed in a 50 mL polystyrene centrifuge tube, followed by the addition of 15 mL of acetonitrile. After shaking for 30 minutes and centrifugation at 9000 rpm for 8 minutes, 1.5 mL of the supernatant was taken for analysis. Chromatographic Conditions: The chromatographic column used was Agilent Poroshell 120 EC-C18 (150 mm x 3.0 mm, 2.7 μm). The mobile phases were A: 0.1% formic acid in 5 mmol/L ammonium formate solution, and B: 0.1% formic acid in acetonitrile. A gradient elution program was implemented with a flow rate of 0.30 mL/min and a column temperature of 40°C. Mass Spectrometry Conditions: Electrospray ionization (ESI) in MRM mode was utilized with specific parameters for Uniconazole, including a precursor ion at m/z 292.0 and product ions at m/z 70.2 and 139.1, with collision energies of 24 eV (He et al., 2023).

### 2.4 Description of uniconazole degradation dynamics

The degradation dynamics of uniconazole in *Ophiopogon japonicus* root tubers and soil were analyzed by plotting residue concentration against time using a first-order kinetic equation (Cao et al., 2015).


$$C_{t}=C_{0}{\text{e}}^{-kt}$$

t12=ln2∕K

in which *C_0_* is the initial concentration of uniconazole, *C_t_* is the concentration of uniconazole at t time (mg/kg), k is the degradation rate constant (d^-1^), and t is the degradation time (d).

### 2.5 Statistical analysis

All data were processed using Excel. Stacked bar charts were created using Origin 2024b. Additionally, principal component analysis (PCA) of the medicinal quality indicators of Ophiopogonis Radix was conducted using Origin 2024b, producing a 2D PCA plot. Bar charts were created using GraphPad Prism 8. Data standardization for medicinal quality indicators of Ophiopogonis Radix was performed using the ‘*Stats*’ package in R. Clustering was conducted with the ‘*Pheatmap*’ package, which generated a circular clustering heatmap using complete linkage and Euclidean distance, along with additional visualizations created through the online platform www.chiplot.online (accessed September 30, 2024) (Jiang et al., 2024). One-way analysis of variance (ANOVA) was performed using SPSS 26.0, with multiple comparisons conducted using Duncan’s new multiple range test.

## 3 Results and discussion

### 3.1 The effects of uniconazole on the growth of *Ophiopogon japonicus*

**Figure 2:**
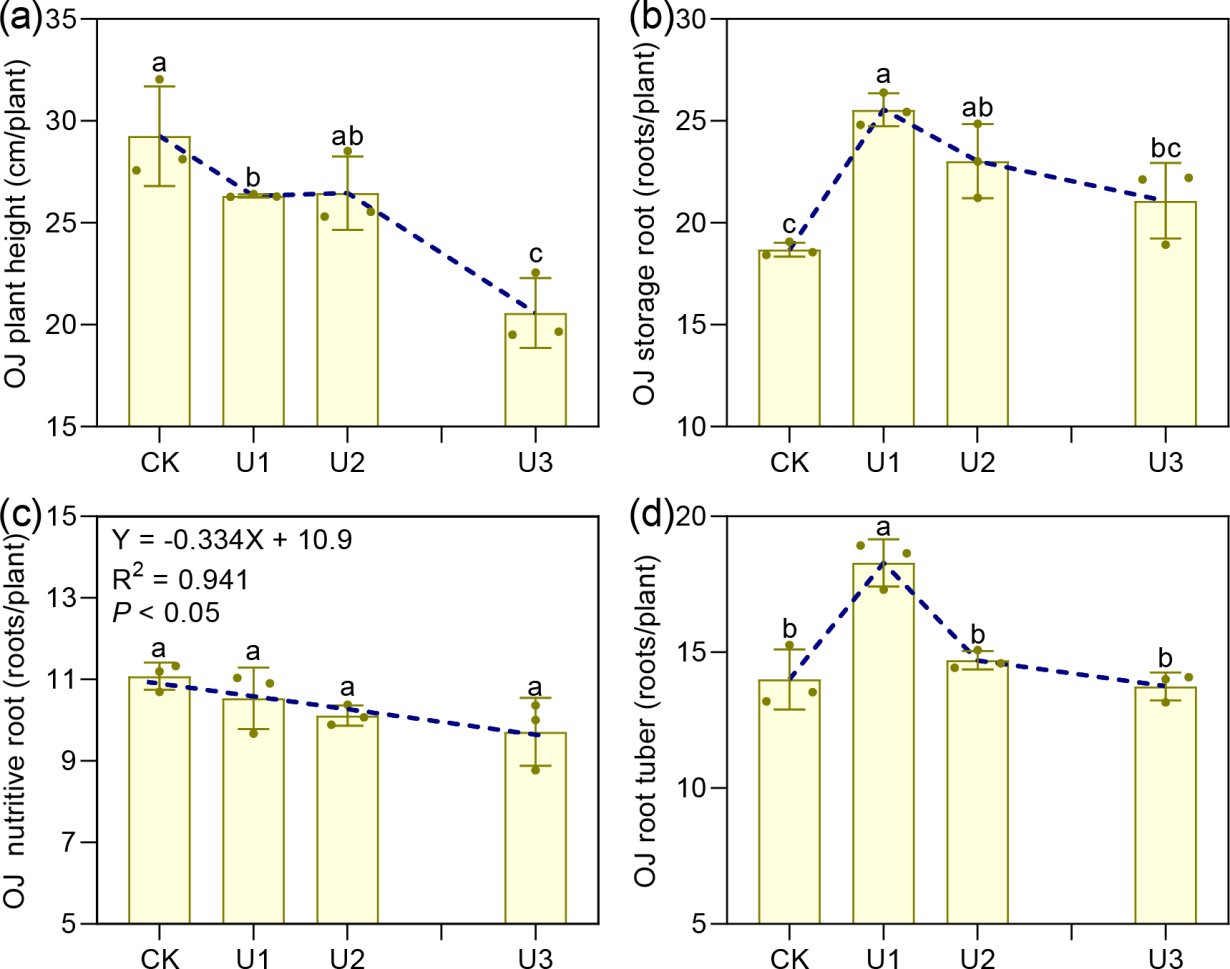
The effects of uniconazole on agronomic traits of *Ophiopogon japonicus* (OJ). **(a)** is OJ plant height, **(b)** is OJ storage root, **(c)** is OJ nutritive root, and **(d)** is OJ root tuber. Different lowercase letters indicate significant differences at the P < 0.05 level. Replicates (n = 3), error bars represent standard deviation (SD).

Under different uniconazole treatments, the height of *Ophiopogon japonicus* (OJ) plants ranged from 20.58 to 29.25 cm (**Figure 2**). Heights in the U1 and U3 groups were reduced by 10.02% and 29.66%, respectively, compared to the control (CK), demonstrating that uniconazole inhibits plant height, especially at higher doses. The number of nutritive roots per plant varied between 9.71 and 11.08, with no marked differences across treatments; however, the regression analysis indicated a decline in nutritive root number as uniconazole concentration increased. For storage roots, counts ranged from 18.68 to 25.55 per plant. Both U1 and U2 treatments showed increases of 36.76% and 23.26%, respectively, relative to CK, while U3 remained comparable to the control. This suggests that uniconazole promotes storage root formation at lower concentrations but may inhibit it at higher levels. Similarly, root tuber numbers per plant ranged from 13.75 to 18.29, with U1 exhibiting a 30.64% increase over CK, whereas U2 and U3 did not differ significantly. These results indicate that uniconazole encourages root tuber swelling at low doses but exerts inhibitory effects as concentration rises.

**Figure 3:**
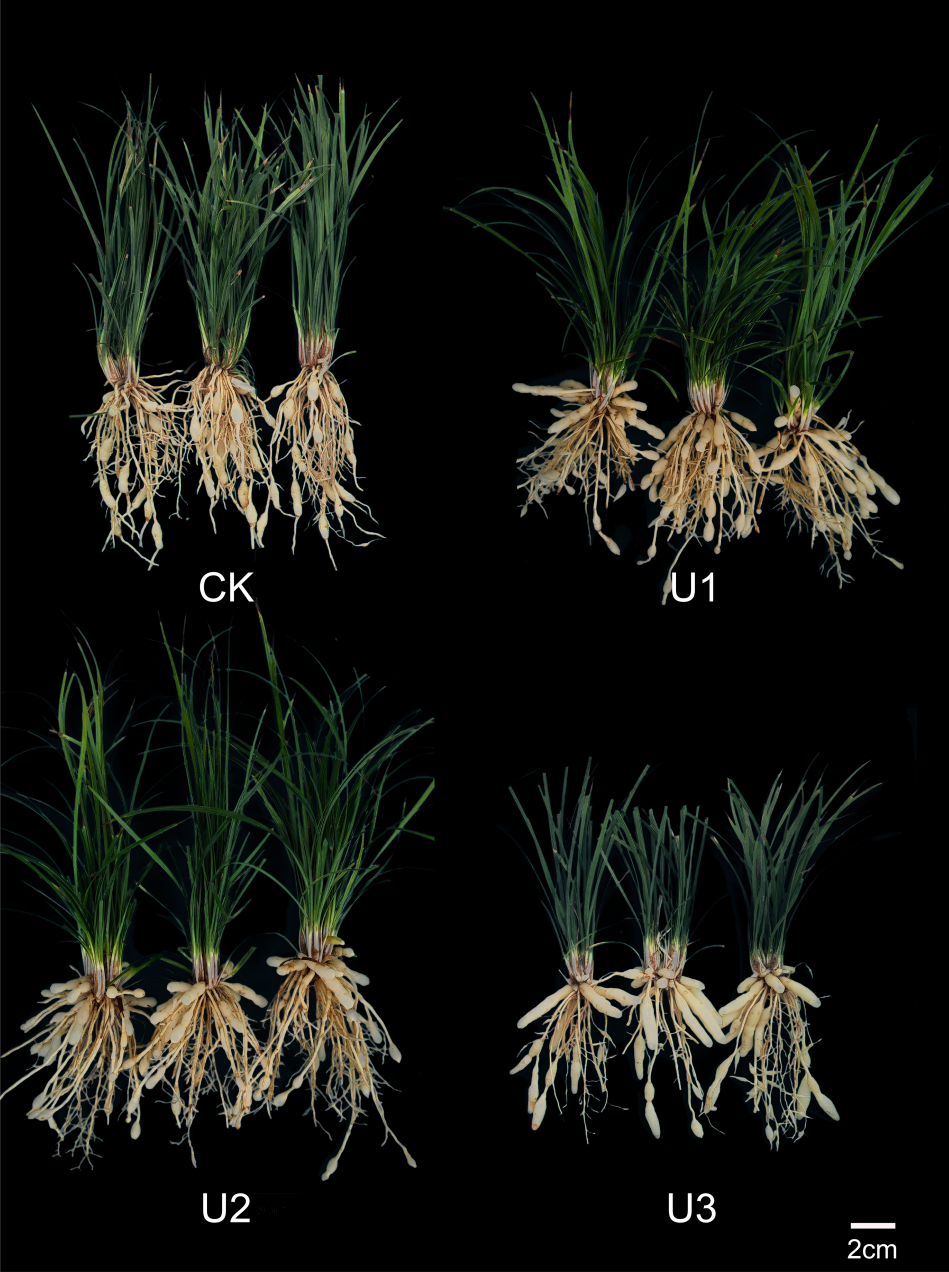
Photographs show the effects of Uniconazole on the whole *Ophiopogon japonicus* plants.

After treatment with uniconazole, the swelling position of the *Ophiopogon japonicus* (OJ) root tuber shifted from the end farthest from the OJ storage root tip to the end closer to the OJ rhizome, or even swelling at the time of formation (**Figure 3**). Notably, the shape of the OJ root tuber changed significantly from spindle-shaped to cylindrical or dumbbell-shaped with rounded ends, and the diameter increased as well. The higher the dose of uniconazole applied, the more pronounced these changes became.

**Figure 4:**
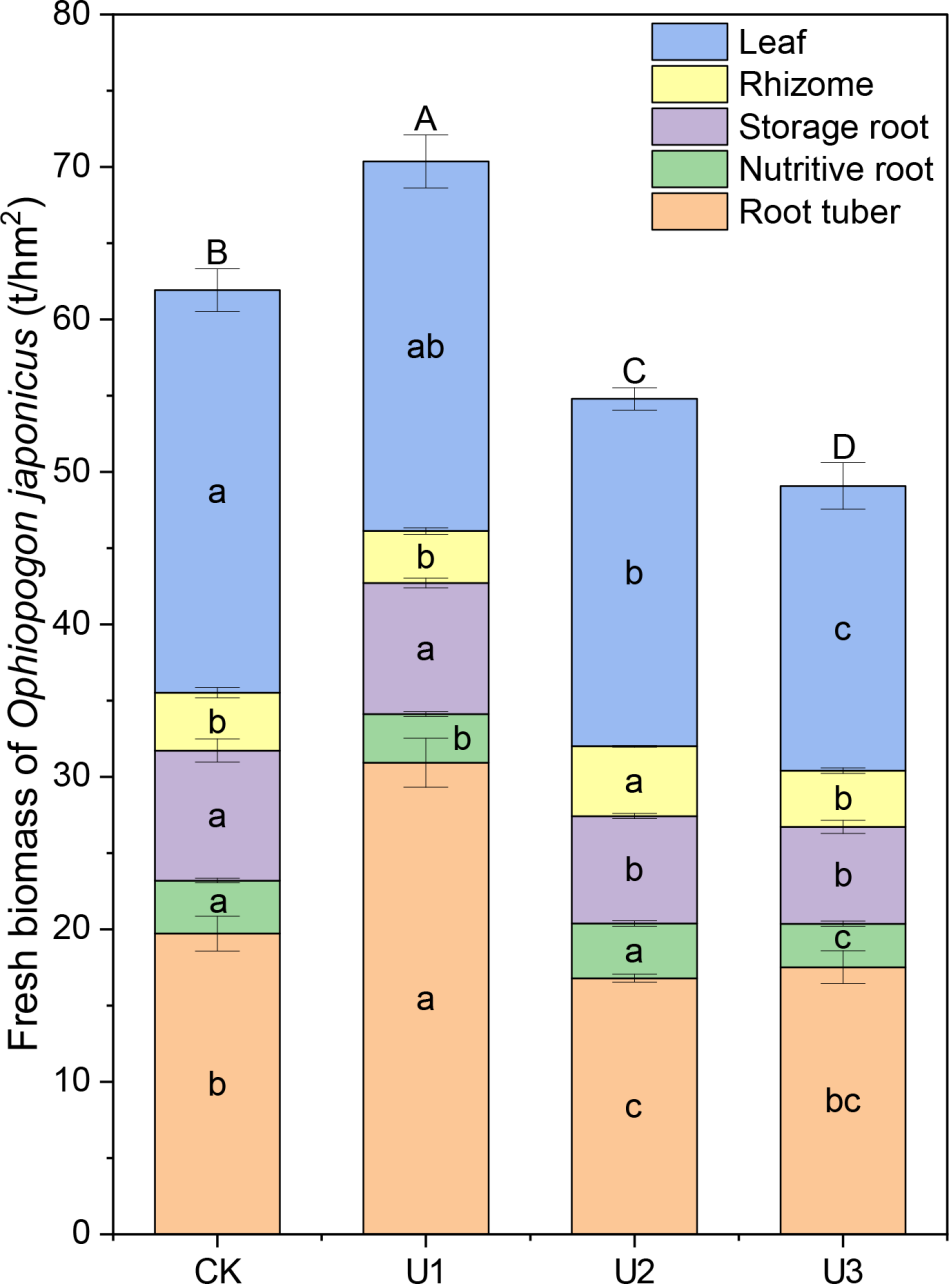
The effects of uniconazole on the accumulation of fresh biomass in *Ophiopogon japonicus* (OJ). Different lowercase letters indicate significant differences at the *P* < 0.05 level. Different uppercase letters indicate significant differences in the total yield of *Ophiopogon japonicus* (sum of different parts) at the *P* < 0.05 level. Replicates (n = 3), error bars represent standard deviation (SD).

Under different uniconazole treatments, the fresh biomass of *Ophiopogon japonicus* (OJ) leaves ranged from 18.68 to 26.40 t/hm² (**Figure 4**). The U2 and U3 treatment groups were significantly lower than the CK (control) group, with reductions of 13.74% and 29.27%, respectively. Although U1 showed an 8.19% decrease compared to CK, this difference was not significant, indicating that uniconazole generally inhibits the fresh biomass of OJ leaves, with high doses leading to stronger inhibition. The fresh biomass of OJ rhizomes ranged from 3.41 to 4.58 t/hm². The U2 treatment group was significantly higher than CK, with an increase of 20.63%, while U1 and U3 did not show significant differences compared to CK. This suggests that medium doses of uniconazole promote the fresh biomass of OJ rhizomes, whereas low and high doses do not significantly affect their biomass. For the fresh biomass of OJ nutritive roots, values ranged from 2.85 to 3.60 t/hm². The U1 and U3 treatments were significantly lower than CK, with reductions of 8.59% and 18.02%, respectively, while U2 did not show a significant difference compared to CK. This indicates that the application of uniconazole generally inhibits the fresh biomass of OJ nutritive roots. The fresh biomass of OJ storage roots ranged from 6.35 to 8.60 t/hm². The U2 and U3 treatment groups were significantly lower than CK, with decreases of 17.44% and 25.54%, respectively, while U1 did not show a significant difference. This suggests that uniconazole overall inhibits the fresh biomass of OJ storage roots, with high doses leading to stronger inhibition. The fresh biomass of OJ root tubers ranged from 16.79 to 30.93 t/hm², with only U1 significantly higher than CK, showing an increase of 56.84%. In contrast, U2 was significantly lower than CK, with a decrease of 14.88%. Although U3 showed an 11.17% reduction compared to CK, this was not significant. This indicates that low doses of uniconazole promote the fresh biomass of OJ root tubers, while medium and high doses generally inhibit their biomass.

Overall, the fresh biomass of the entire *Ophiopogon japonicus* (OJ) plant ranged from 49.08 to 70.37 t/hm². Only U1 was significantly higher than CK, with an increase of 13.62%. In contrast, U2 and U3 were significantly lower than CK, with reductions of 11.54% and 20.76%, respectively. This shows that low doses of uniconazole promote the fresh biomass of the entire OJ plant, while medium and high doses generally inhibit its biomass.

### 3.2 The effects of uniconazole on the hormonal changes in different plant parts of *Ophiopogon japonicus*

**Figure 5:**
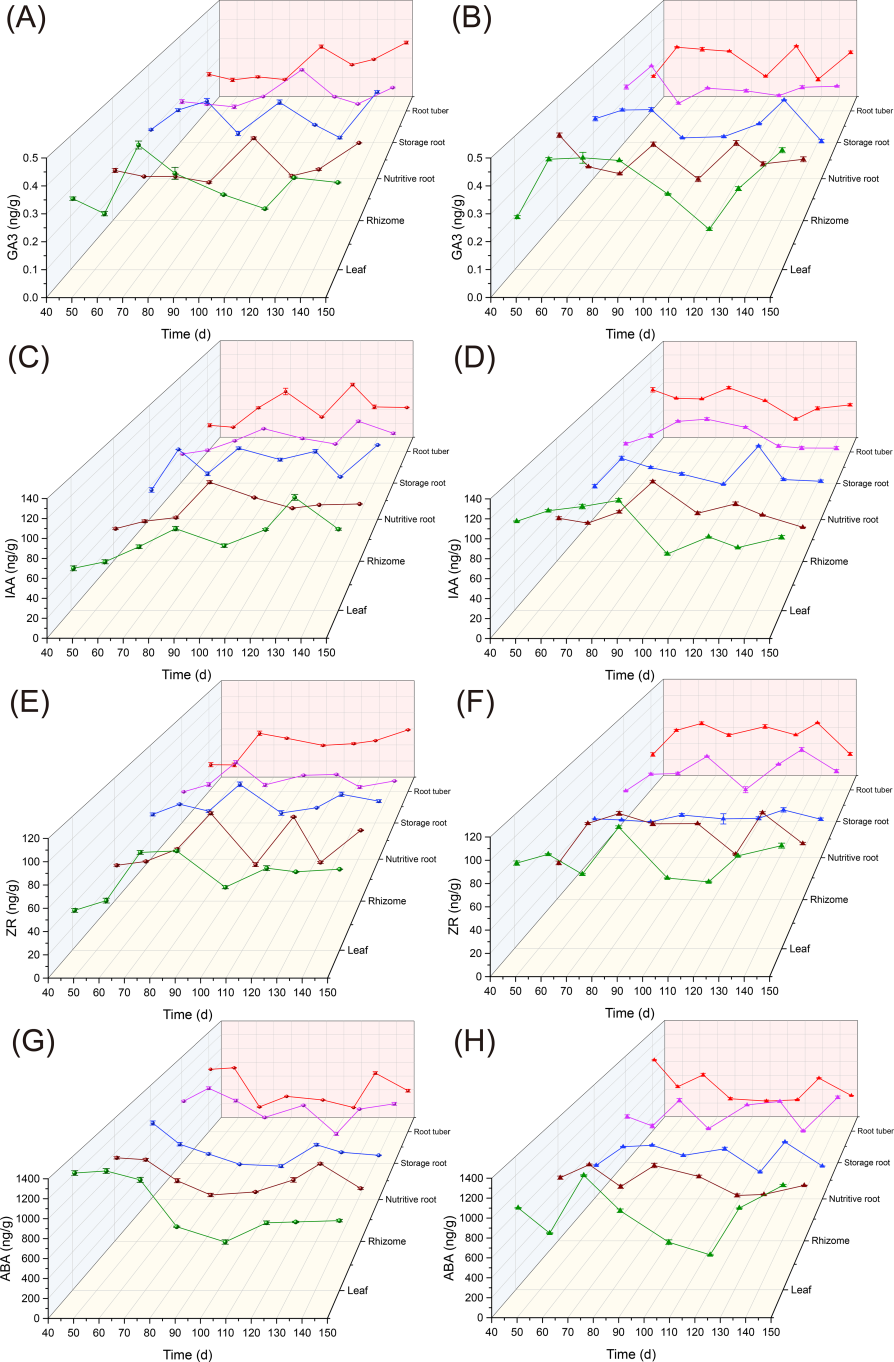
The effects of uniconazole on hormonal changes in different plant parts of *Ophiopogon japonicus*. **(A)** GA_3_ content in the CK (control) group; **(B)** GA_3_ content in the U3 treatment group; **(C)** IAA content in the CK group; **(D)** IAA content in the U3 group; **(E)** ZR content in the CK group; **(F)** ZR content in the U3 group; **(G)** ABA content in the CK group; **(H)** ABA content in the U3 group. GA_3_, Gibberellic Acid 3; IAA, Indole-3-Acetic Acid; ZR, Zeatin Riboside; ABA, Abscisic Acid. Replicates (n = 3); error bars represent standard deviation (SD).

According to previous studies, uniconazole application on *Ophiopogon japonicus* (OJ) root tubers shows distinct growth phases: an initial tuber expansion peak between days 41 and 54 (T1), a slow expansion period between days 55 and 103 (T2), and a second peak expansion phase between days 104 and 150 (T3) (Wu et al., 2018). The effects of uniconazole on hormone levels in different parts of *Ophiopogon japonicus* over time are shown in **Figure 5**. Hormone accumulation in various plant parts during different stages of tuber enlargement is presented in **Supplementary Table S1**.

For Gibberellic Acid 3 (GA_3_) levels, in the control (CK) group, relative growth rates in *Ophiopogon japonicus* (OJ) leaves during T1, T2, and T3 were -1.83%, 0.60%, and 0.32%, respectively. After uniconazole treatment, relative growth rates increased in T1 and T3, reaching 5.71% and 0.97%, while T2 showed a decline to -0.77%. In the rhizomes, CK group growth rates were -0.99%, 1.28%, and -0.13% in T1, T2, and T3, respectively, whereas post-treatment values dropped to -3.53% and -0.54% in T1 and T2, with a rise in T3 to 0.83%. The OJ nutritive root showed relative growth rates of 2.58%, 0.22%, and 0.27% in T1, T2, and T3 in CK, while post-treatment values decreased to 1.04%, -1.00%, and -0.24%, respectively. Storage roots in CK recorded rates of -0.48%, 1.31%, and -0.60% during T1, T2, and T3, shifting to 2.48%, -0.80%, and 0.18% post-treatment. In root tubers, CK growth rates were -1.28%, 1.49%, and 0.13% for T1, T2, and T3, increasing to 4.72% and 1.12% in T1 and T3, with T2 declining to -1.25% after uniconazole application.

For Indole-3-Acetic Acid (IAA) levels, *Ophiopogon japonicus* (OJ) leaves in the CK group showed relative growth rates of 1.11%, 0.59%, and 0.48% across T1, T2, and T3, while treatment with uniconazole led to declines in T1 and T2 to 0.86% and -1.16%, and an increase in T3 to 0.55%. In the rhizomes, CK group rates were 1.58%, 0.95%, and -0.23% across T1, T2, and T3, while post-treatment values fell across all periods to -0.92%, 0.47%, and -0.73%. OJ nutritive roots showed CK growth rates of 6.68%, -0.32%, and 0.47% for T1, T2, and T3, decreasing to 4.67%, -1.12%, and 0.17% after treatment. Storage root CK values were 0.93%, 0.63%, and 0.23% across T1, T2, and T3, shifting post-treatment to 1.41%, 0.33%, and -0.98% in T1, T2, and T3. In root tubers, CK rates were -0.57%, 0.71%, and 0.52% for T1, T2, and T3, with uniconazole treatment resulting in a decline across all periods to -1.13%, -0.09%, and -0.18%.

For Zeatin Riboside (ZR) levels, CK treatment in *Ophiopogon japonicus* (OJ) leaves showed relative growth rates of 1.69%, 0.49%, and 0.54% for T1, T2, and T3, while uniconazole treatment resulted in declines for T1 and T2 to 0.76% and -0.59%, and an increase in T3 to 0.80%. In rhizomes, CK rates were 0.79%, -0.17%, and 1.41% for T1, T2, and T3, while treatment led to increases in T1 and T2 to 5.62% and 0.00%, and a decline in T3 to -0.65%. OJ nutritive roots in CK showed rates of 1.58%, -0.36%, and 0.49% in T1, T2, and T3, with post-treatment declines in T1 and T3 to -0.23% and -0.01%, and a T2 increase to 0.05%. In storage roots, CK rates were 1.59%, 0.44%, and -0.27% for T1, T2, and T3, while uniconazole treatment shifted values to 3.34%, -0.81%, and 0.98%. Root tuber CK group values were -0.06%, 1.13%, and 0.62%, with treatment leading to a rise in T1 to 3.99% and declines in T2 and T3 to 0.13% and -1.21%.

For Abscisic Acid (ABA) levels, CK-treated leaves showed relative growth rates of 0.13%, -1.84%, and 0.78% in T1, T2, and T3, while uniconazole application resulted in declines in T1 to -2.84% and increases in T2 and T3 to -0.35% and 1.67%. Rhizome CK rates were -0.18%, 1.02%, and 0.15% for T1, T2, and T3, with uniconazole increasing values in T1 and T2 to 1.44% and -0.35%, and a decrease in T3 to -0.32%. Nutritive roots in CK showed rates of -2.50%, -1.03%, and 0.60%, with treatment leading to increases in T1 and T2 to 3.39% and -0.08%, and a T3 drop to -0.90%. Storage roots in CK were 1.45%, -0.53%, and 0.06% in T1, T2, and T3, with uniconazole bringing values down in T1 to -1.77%, and up in T2 and T3 to 0.93% and 0.26%. Root tuber CK group rates were 0.18%, -1.43%, and 0.55% across T1, T2, and T3, with uniconazole treatment leading to T1 and T3 increases to -3.62% and 0.36%, and a T2 drop to -0.80%.

In summary, compared to the control group (CK), the levels of Gibberellic Acid 3 (GA_3_) in various parts of *Ophiopogon japonicus* (OJ) (excluding the OJ nutritive root) primarily promoted growth during T1 and T3, while inhibiting growth during T2. For the OJ nutritive root, GA_3_ showed inhibitory effects across all three periods. Indole-3-Acetic Acid (IAA) consistently exhibited a predominant inhibitory effect in all parts of OJ during the three periods. Zeatin Riboside (ZR) primarily promoted growth in T1 but shifted to an inhibitory effect in T2 and T3. Abscisic Acid (ABA) promoted growth in T2, while it had an inhibitory effect during T1 and T3.

### 3.3 The effects of uniconazole on the characteristics of Ophiopogonis Radix

**Figure 6:**
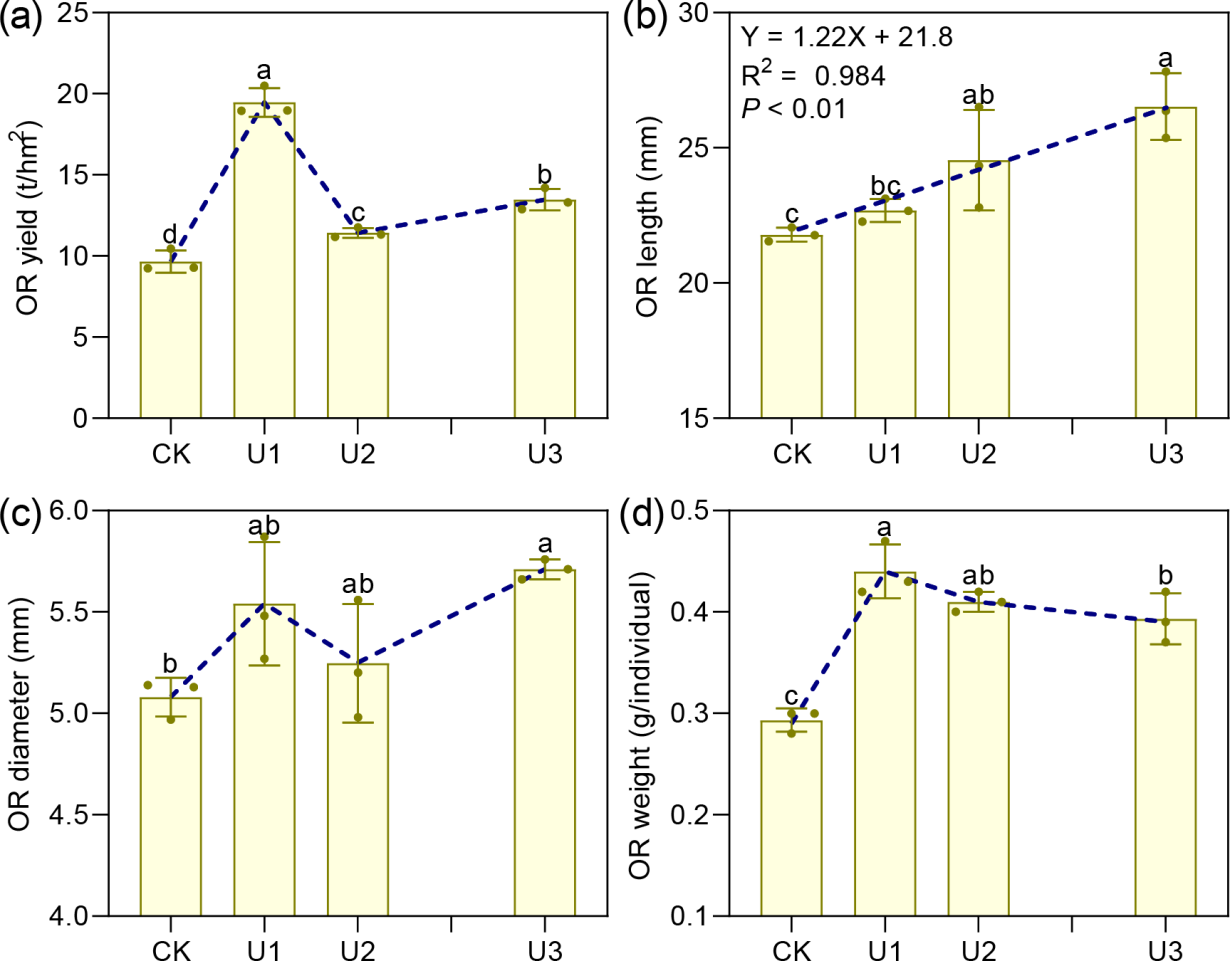
The effects of Uniconazole on the yield and morphology of *Ophiopogon japonicus* (OJ) root tubers. **(a)** is OR yield, **(b)** is OR length, **(c)** is OR diameter, and **(d)** is OR wight. *Ophiopogon japonicus* yield refers to the fresh weight of the root tubers. Ophiopogonis Radix (OR) denotes the root tubers processed for medicinal use. Replicates (n = 3), error bars representl standard deviation (SD).

**Figure 7:**
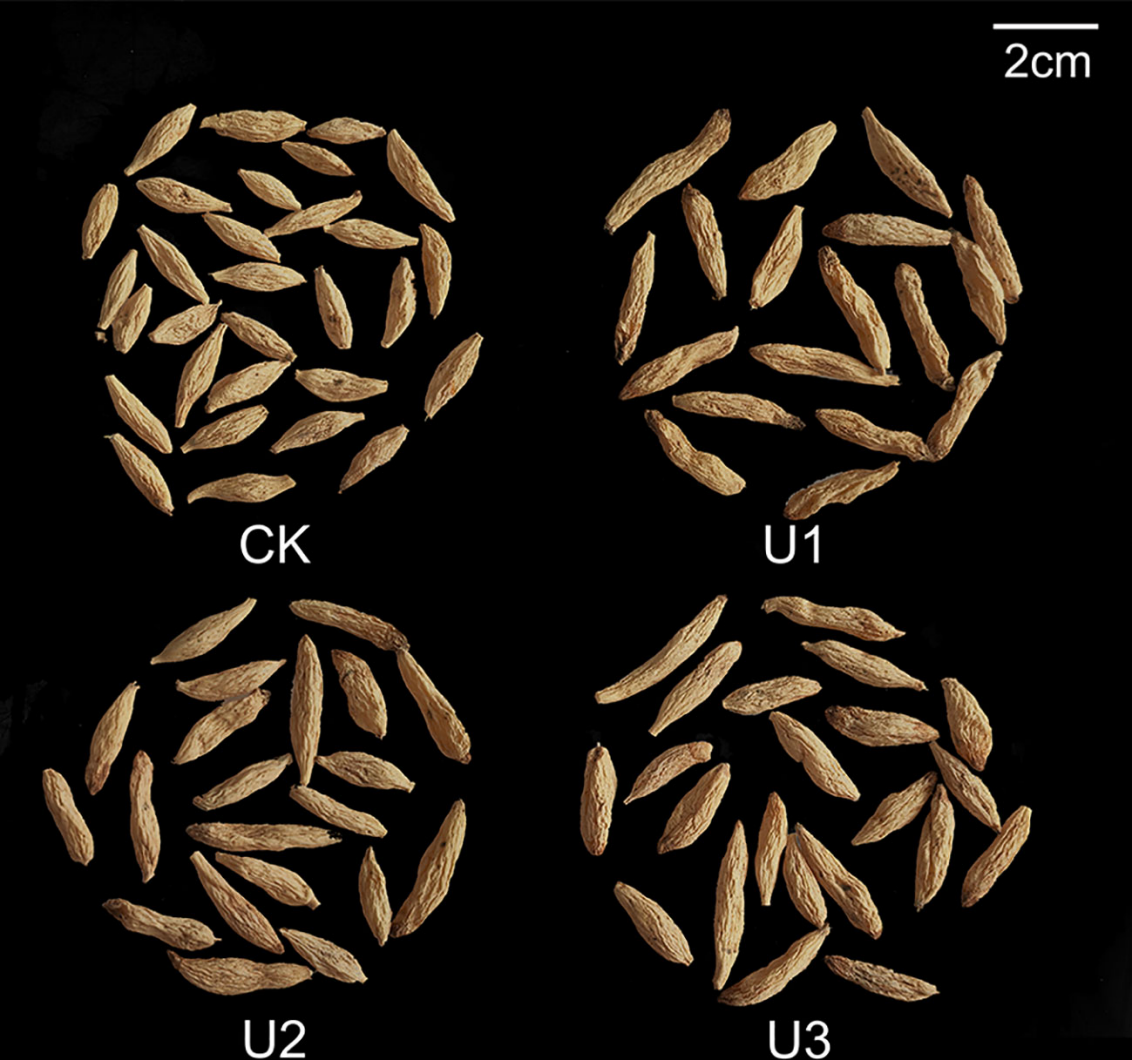
Photographs show the effects of Uniconazole on the morphology and size of Ophiopogonis Radix.

Under varying uniconazole treatments, the yield of Ophiopogonis Radix (OR) ranged from 9.65 to 19.46 t/hm², with all treatments increasing yield compared to the control (CK) by 101.59%, 18.27%, and 39.38%, respectively (**Figure 6**). This demonstrates that uniconazole promotes OR yield in a dose-dependent manner, characterized by a low-dose stimulation and high-dose inhibition, with U1 producing the greatest yield enhancement. The shape of OR is shown in **Figure 7**. OR length varied from 21.79 to 26.52 mm, with U2 and U3 treatments extending length by 12.64% and 21.75% over CK, while the 4.13% increase seen in U1 was not statistically meaningful. Overall, uniconazole favors OR elongation, particularly at higher doses, consistent with a linear regression model. Diameter measurements ranged between 5.08 and 5.71 mm, with U3 surpassing CK by 12.36%, indicating a general trend of increased diameter formation at higher uniconazole concentrations. Dry weight of single OR ranged from 0.29 to 0.44 g, with all treatments boosting this metric by 49.53%, 38.94%, and 32.46%, respectively, relative to CK. This suggests uniconazole effectively enhances OR weight, especially at lower doses where the impact is most pronounced.

### 3.4 The effects of uniconazole on the medicinal quality indicators of Ophiopogonis Radix

**Figure 8:**
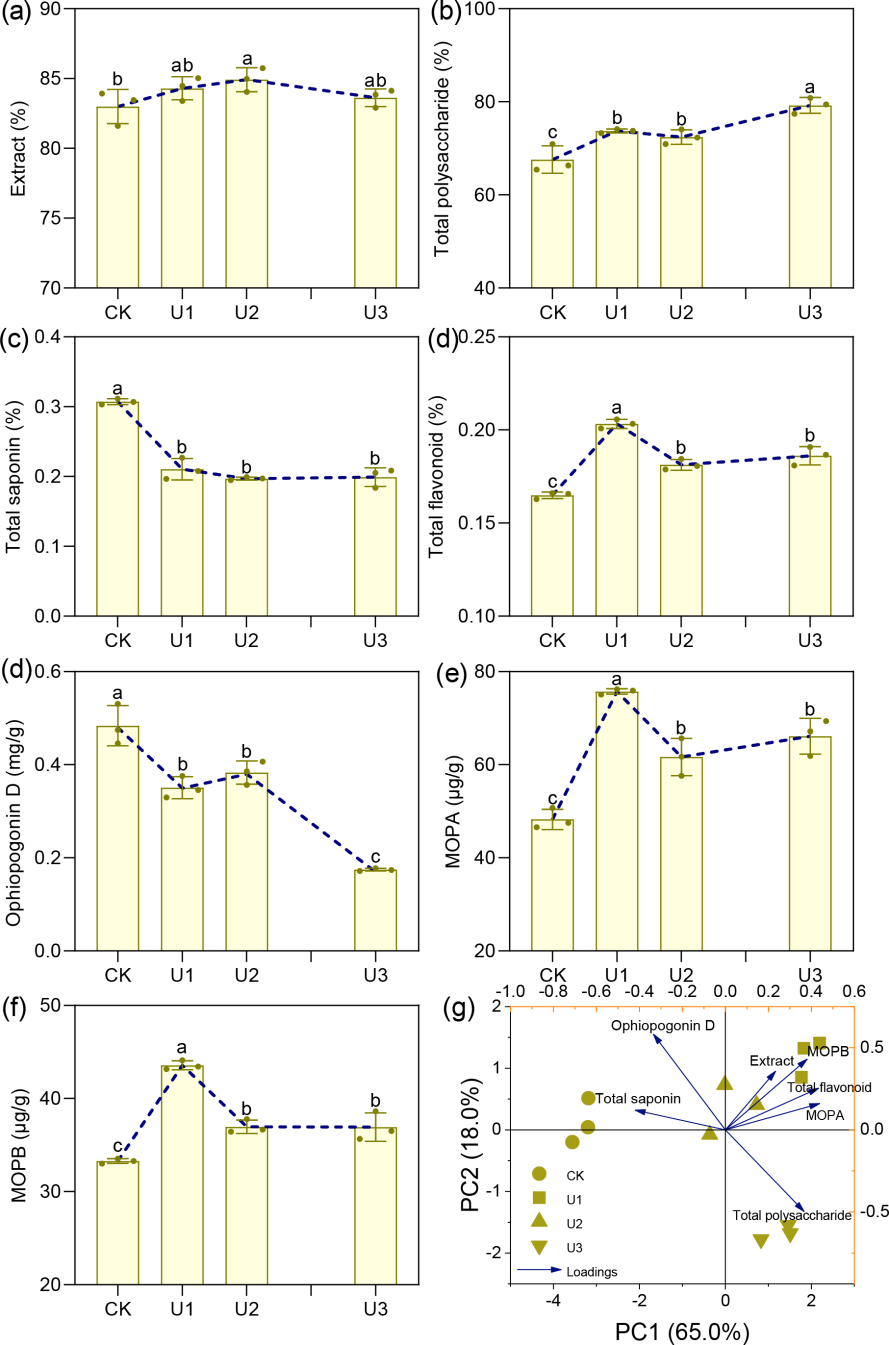
The effects of uniconazole on the medicinal quality indicators of Ophiopogonis Radix (OR). MOPA means Methylophiopogonanone **(a)**, MOPB means Methylophiopogonanone **(b)** Figure **(g)** is the principal component analysis (PCA) of all medicinal quality indicators. The score plot is displayed on the left y-axis and the bottom x-axis, while the loading plot is shown on the right y-axis and the top x-axis. Replicates (n = 3), error bars represent standard deviation (SD).

Under varying concentrations of uniconazole treatment, the extract content of Ophiopogonis Radix ranged from 83.00% to 84.92%, with the U2 treatment group showing a significant 2.32% increase compared to the CK (**Figure 8**). Total polysaccharide content exhibited a consistent upward trend with increasing uniconazole dosage, with treatments U1 to U3 showing significant improvements of 7.21% to 17.25% over CK. Total saponins and Ophiopogonin D—two key pharmacologically active constituents in Ophiopogonis Radix—both showed a consistent decline as uniconazole concentration increased. Total saponin levels decreased by 31.51% to 35.90%, indicating that uniconazole suppresses saponin accumulation across all tested doses. Similarly, Ophiopogonin D levels declined significantly in all treatment groups, with reductions ranging from 20.80% to 63.94%, and the degree of suppression intensified with higher uniconazole application. Flavonoid compounds, including total flavonoids, MOPA, and MOPB, followed a similar trend: an initial increase followed by a decline. The U1 treatment was the most effective, significantly increasing total flavonoids by 23.29%, MOPA by 56.94%, and MOPB by 30.92% compared to CK. In summary, uniconazole treatment can enhance certain quality indicators of Ophiopogonis Radix, such as polysaccharides and flavonoids, particularly at lower concentrations. However, it significantly reduces the content of key active ingredients like total saponins and Ophiopogonin D (Commission C P, 2020), with greater reductions observed at higher doses. This highlights the need for careful optimization of uniconazole use to balance yield improvement with the preservation of medicinal quality.

Principal Component Analysis (PCA) was conducted on the quality indicators and medicinal properties of Ophiopogonis Radix (**Figure 5g**). The first principal component (PC1) accounted for 65.00% of the variance, while the second (PC2) accounted for 18.00%, together explaining 83.00% of the variance. This suggests that PC1 and PC2 encapsulate most of the quality and medicinal indicator information of Ophiopogonis Radix, providing a basis for evaluating quality and medicinal properties across the four treatment groups. The treatments displayed significant differences, with U1 and U1 clustering together, and CK and U3 each forming separate clusters. Medicinal quality indicators were grouped as follows: Extract, Total flavonoid, Methylophiopogonanone A, and Methylophiopogonanone B formed one group, Total saponin and Ophiopogonin D formed another, while Total polysaccharide was categorized separately.

**Figure 9:**
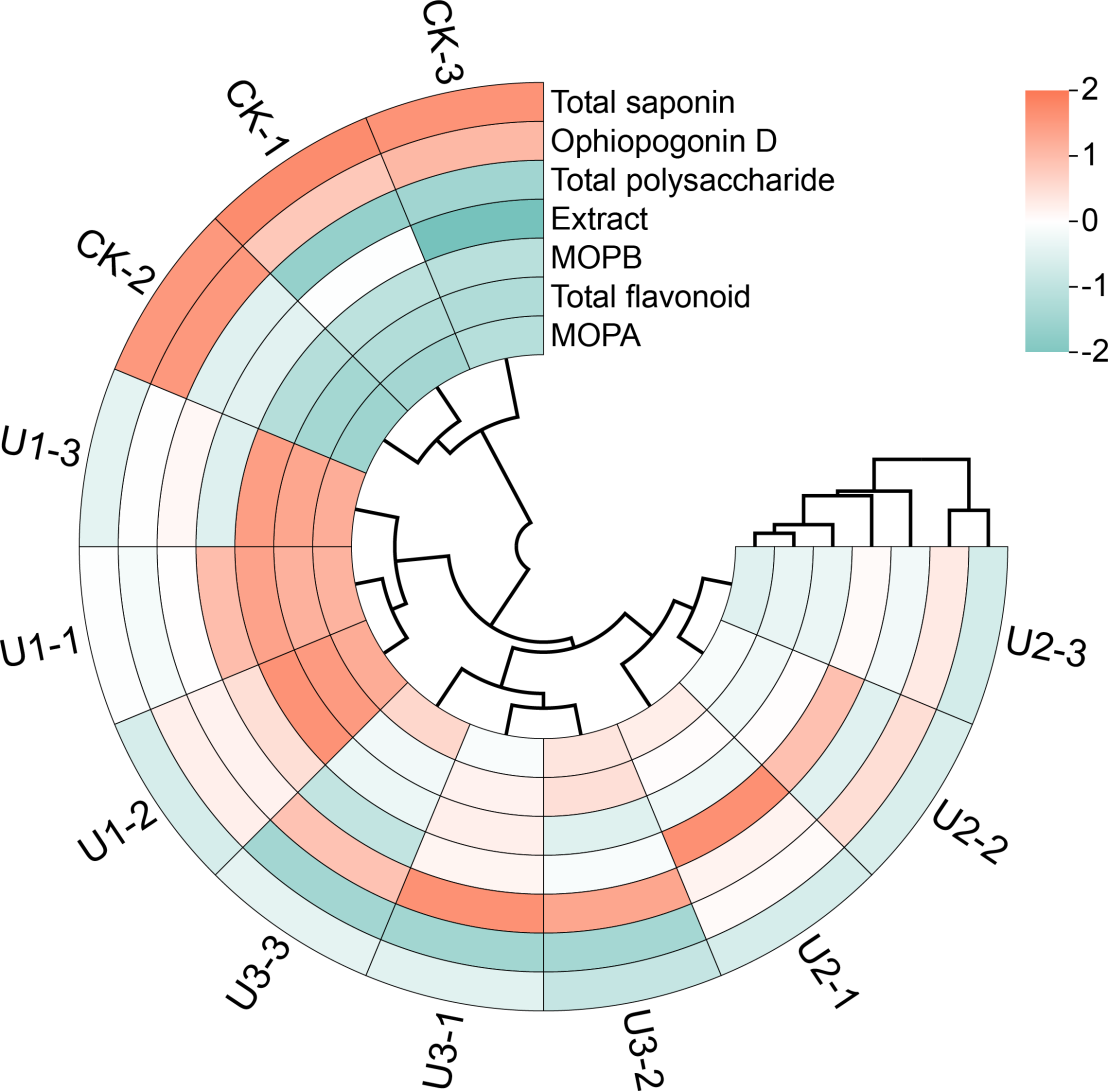
Circular clustered heatmap of standardized medicinal quality indicators in Ophiopogonis Radix (OR). The heatmap displays the standardized values (Z-scores) of selected medicinal quality indicators across different samples. The color scale represents Z-scores, with red indicating higher-than-average values and green indicating lower-than-average values (range: -2 to +2).

According to the circular clustering analysis of the medicinal quality indicators of Ophiopogonis Radix, each treatment group clustered separately (**Figure 9**). The first cluster was CK, which showed the highest levels in the medicinal quality indicators Total Saponin and Ophiopogonin D, but the lowest in Total Flavonoid, Methylophiopogonanone A, and Methylophiopogonanone B. The second cluster, U1, showed the highest levels for Total Flavonoid, Methylophiopogonanone A, and Methylophiopogonanone B. The third cluster, U3, had the highest level of Total Polysaccharide. The fourth cluster, U2, exhibited the highest levels in Extract. Overall, the three Uniconazole-treated groups (U1, U2, and U3) were clustered together, distinguishing them from the CK group.

### 3.5 The residual effects of uniconazole on Ophiopogonis Radix and soil

**Figure 10:**
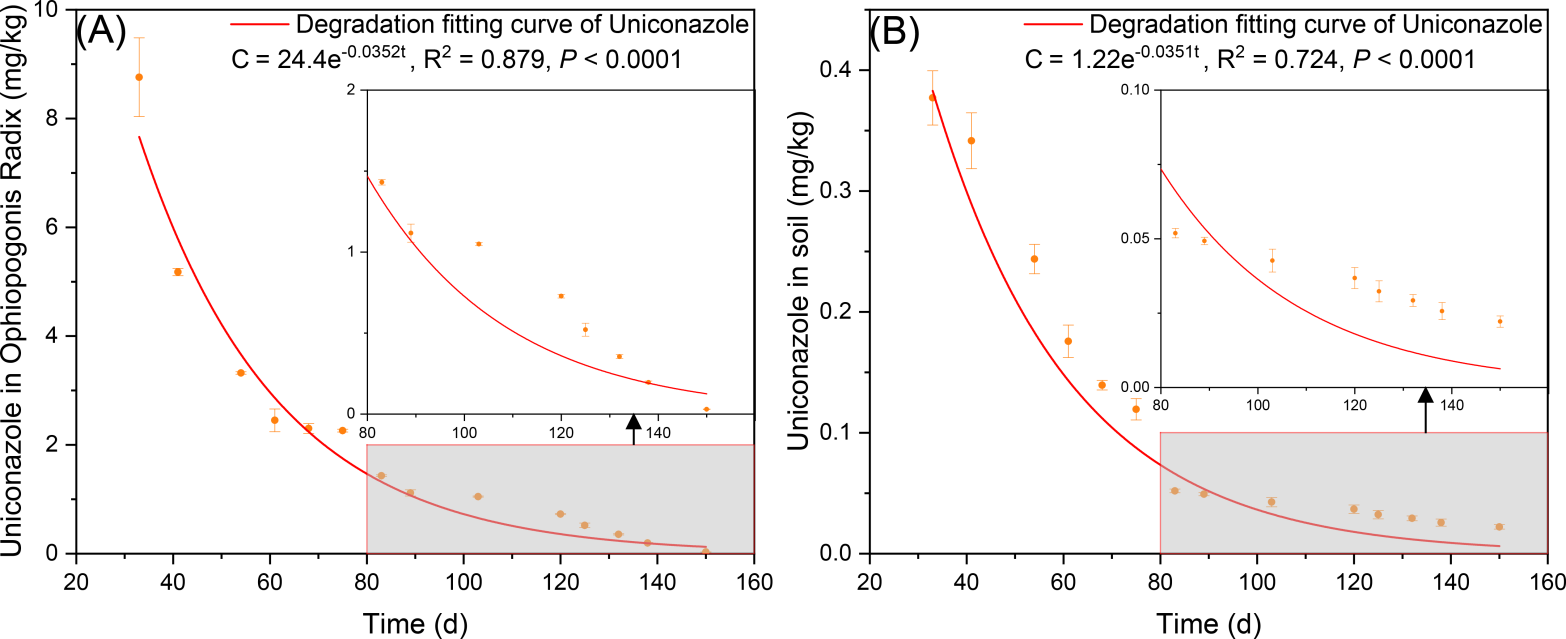
The degradation curves of uniconazole, **(A)** is Ophiopogonis Radix (OR), and **(B)** is the soil. *C* is the concentration of uniconazole at t time (mg/kg), t is the degradation time (d). Replicates (n = 3), error bars represent standard deviation (SD).

Under the U3 treatment (30 kg/hm²), the degradation fitting curve for uniconazole in Ophiopogonis Radix indicates an initial theoretical residue of 24.4 mg/kg, with a model of *C* = 24.4e^-0.0352t^. The half-life *T_1/2_* = 19.69 d, with 99% degradation occurring at *T_0.99_* = 130.83 d, and a correlation coefficient R^2^ = 0.879 (**Figure 10**). Uniconazole residue in Ophiopogonis Radix showed a negative correlation with sampling time. For soil, under the same U3 treatment, the degradation fitting curve indicates an initial theoretical residue of 1.22 mg/kg, following *C* = 1.22e^-0.0268t^. The half-life *T_1/2_* = 19.75 d, with 99% degradation at *T_0.99_* = 131.20 d, and a correlation coefficient R^2^ = 0.724. Similarly, uniconazole residue in soil decreased negatively over time. At harvest (150 days after Uniconazole application), both Ophiopogonis Radix and soil residue levels were below 0.05 mg/kg. Uniconazole degradation reached 99.88% in Ophiopogonis Radix.

## 4 Discussion

### 4.1 Dual-effects of uniconazole dosage

Uniconazole is a plant growth regulator that functions similarly to paclobutrazol. It inhibits vertical elongation in crops, leading to shorter plant heights while promoting lateral growth, which results in thicker stems and roots. It is commonly used in crops such as rice (Liu et al., 2024) and wheat (Peake et al., 2020) to increase tillering, control height, and improve resistance to lodging. Additionally, uniconazole treatment has been shown to reduce the height of ornamental plants like petunias (Karimi and Ahmadi, 2019) and zinnias (Alami and Karimi, 2020), achieving a more compact growth form that enhances their aesthetic appeal. Uniconazole is a triazole-based plant growth regulator that influences plant morphology and development through multiple molecular mechanisms. Its primary mode of action involves modulating hormonal pathways, altering the expression of genes related to growth and stress responses, and reshaping metabolic processes. Collectively, these mechanisms lead to changes in plant height, root development, stress tolerance, and other morphological traits. One of the most well-documented effects of uniconazole is its inhibition of gibberellin (GA) biosynthesis, resulting in reduced GA levels and consequently inducing a dwarfing effect in plants (Qin et al., 2022; Zhang et al., 2022b). Transcriptomic analyses have also shown that uniconazole affects the expression of genes involved in lignin biosynthesis, leading to decreased lignin content and inhibited hypocotyl elongation (Zhang et al., 2022b). In addition, uniconazole alters the levels of other key hormones such as abscisic acid (ABA) and auxin, both of which play essential roles in embryonic development and root formation (Han et al., 2017; Chen et al., 2022). Our study found that uniconazole significantly affects the height of *Ophiopogon japonicus* (OJ); higher concentrations resulted in more pronounced dwarfing effects, with a maximum reduction in height of 29.66%. Concurrently, the fresh biomass of OJ leaves decreased significantly, with a maximum reduction of 29.27%. The observed decreases in plant height and leaf biomass may be related to the “polar transport” of endogenous hormones like Indole-3-Acetic Acid (IAA) within the plants. Similar findings have been reported in studies involving wheat and barley (Jacobs, 1972; Fischer et al., 1997; Wong et al., 2023). Furthermore, uniconazole treatment promotes root growth (Feng et al., 2020), and increases the number of root tips (Zhou et al., 2021). In tuberous crops like potatoes and radishes, the use of this regulator has led to significant yield increases (Duan et al., 2020; Xu et al., 2022), aligning with our results. Our findings indicate that low doses of uniconazole can significantly enhance the quantity and fresh biomass of OJ storage roots and root tubers, thereby improving the yield of Ophiopogonis Radix. While uniconazole can foster desirable traits such as reduced plant height, excessive use may hinder growth and reduce biomass, highlighting the importance of careful dosage management. This “low-dose stimulation, high-dose inhibition” phenomenon—known as hormesis—is underpinned by a range of cellular stress response mechanisms involving complex signaling pathways and molecular interactions. These include cell signaling cascades, protein quality control systems, antioxidant defenses, cellular protection mechanisms, as well as the regulation and repair of gene expression (Mattson, 2008; Schirrmacher, 2021).

Low levels of chemical stress can promote plant growth by increasing chlorophyll content, enhancing carbon dioxide fixation, and stimulating the synthesis of cytokinins. These positive effects are mediated by signaling pathways such as inositol phosphate and MAPK cascades, which are activated in response to mild stress conditions (Kovács-Bogdán et al., 2010). In contrast, high levels of stress agents can inhibit plant growth. For example, in *Arabidopsis*, elevated concentrations of extracellular nucleotides like ATPγS and ADPβS suppress root hair development through signaling pathways involving nitric oxide (NO) and reactive oxygen species (ROS). This demonstrates a clear dose-dependent dual effect—while low concentrations of these nucleotides stimulate growth, higher concentrations activate inhibitory pathways (Clark et al., 2010). A similar pattern has been observed in animal systems. Low doses of methamphetamine (METH) enhance synaptic plasticity and memory by modulating proteins such as Rac1 and Cdc42. However, high doses lead to synaptic degeneration and cognitive impairment (Ding et al., 2022). These examples collectively underscore the hormetic nature of biological responses, where dose determines whether the effect is beneficial or harmful. In this context, low levels of stress can trigger beneficial cellular responses, enhancing resilience and adaptability. However, it is important to note that excessive or improperly managed stress can lead to adverse effects, highlighting the delicate balance required to maintain optimal cellular function (Mattson, 2008).

### 4.2 Uniconazole-induced hormonal reconfiguration in *Ophiopogon japonicus*

Based on the comparative study of high dose (U3) uniconazole treatment during the swelling phase of *Ophiopogon japonicus* (OJ) with the control (CK), we tracked the dynamic changes of four major endogenous hormones in OJ root tubers. This analysis revealed the relationship between hormonal changes and the swelling process of OJ root tubers. During the active swelling phase, the relative growth rates of endogenous hormones were relatively high; as this phase slowed, the growth rates decreased. These hormones primarily promote the division and differentiation of adventitious root meristem cells, with their growth patterns reflecting the swelling dynamics of OJ root tubers. In terms of Gibberellic Acid 3 (GA_3_), uniconazole acts as a biosynthesis inhibitor, significantly affecting GA_3_ production across different plant parts by inhibiting key enzymes in the GA biosynthesis pathway, particularly cytochrome P450 monooxygenases essential for active gibberellins (Rademacher, 2000). This aligns with our results, as we observed a decline in the relative growth rates of GA_3_ in all parts of OJ during the T2 period, indicating that uniconazole treatment suppresses GA_3_ biosynthesis. In T1 and T3 periods, the GA_3_ growth rates in OJ rhizome and nutritive roots decreased, affecting GA_3_ biosynthesis. Similar results have been found in studies involving tomatoes (Kataoka et al., 2008), where uniconazole treatment led to reduced GA_3_ levels, impacting stem elongation, the development of nutritional organs, and overall plant growth. However, during T1 and T3, the GA_3_ growth rates increased in the leaves, storage roots, and root tubers of OJ, suggesting that uniconazole promotes GA_3_ biosynthesis in these areas. Research shows that increased GA_3_ can enhance the number of lateral roots and improve rooting rates, particularly at lower concentrations (Tanimoto, 2012; Yuan and Xiao, 2014). However, excessive use may alter root morphology. Our findings are consistent with this, as uniconazole application significantly increased the quantity and yield of OJ storage roots and root tubers, demonstrating a phenomenon where low concentrations promote growth while high concentrations inhibit it. The application of uniconazole also increased GA_3_ levels; however, excessive GA_3_ and its interactions with other hormones can change root morphology (Pelizza et al., 2009; Han et al., 2020). We observed that uniconazole-treated OJ root tubers often exhibited cylindrical or dumbbell shapes, with more significant changes at high doses. Additionally, in OJ leaves, increased GA_3_ during T1 and T3 periods was linked to the formation and accumulation of storage roots and root tubers. Studies on crops like tobacco and Cape Gooseberry (Manoharlal et al., 2018; El-Tohamy et al., 2023) have shown similar results, indicating that increased GA_3_ in leaves enhances chlorophyll content and photosynthetic rates, thereby improving overall crop efficiency.

In terms of Indole-3-Acetic Acid (IAA), our findings showed a decreasing trend in the relative growth rates of IAA across all parts of *Ophiopogon japonicus* (OJ) during T1, T2, and T3 periods, suggesting that uniconazole treatment significantly affects IAA synthesis. This is consistent with research on cotton and soybean (Zhou et al., 2021; Chen et al., 2022). IAA is known to promote root length, lateral root formation, and significantly enhance root biomass (Agnnas and Abayathunga, 2023; Ganesh et al., 2024). However, in our study, the formation of fresh biomass in OJ storage roots exhibited an overall inhibitory effect, with high doses leading to stronger inhibition (up to 25.54%). This closely relates to the significant reduction in IAA synthesis due to uniconazole application, which also affected the length and shape changes of OJ storage roots and root tubers. The formation of fresh biomass in OJ root tubers exhibited a Hormesis pattern (Wan et al., 2024), reflecting IAA’s dual role—promoting growth at low concentrations while inhibiting it at high concentrations (Agnnas and Abayathunga, 2023). IAA biosynthesis can also impact gene expression related to root development (Li et al., 2024), underscoring the need for cautious application of uniconazole in agricultural practices.

In terms of Zeatin Riboside (ZR), following uniconazole treatment, the relative growth rates of ZR in *Ophiopogon japonicus* (OJ) rhizome, storage root, and root tuber increased during T1. ZR, a type of cytokinins, plays a crucial role in promoting root growth and development. ZR enhances lateral root formation, stimulates root meristem activity, and increases root length and density (Jabir et al., 2017; Wang and Yang, 2021; Al-Mamun et al., 2024), aligning with our findings. Our study indicates that uniconazole promotes rooting in OJ rhizomes, significantly increasing the quantity and biomass of storage roots and root tubers. During T2, the relative growth rates of ZR in OJ rhizome and nutritive roots also increased. As the nutrient transport and absorption organs for OJ, the enhanced ZR synthesis in these areas correlates with the increased biomass and material accumulation in storage roots and root tubers. Existing research has shown that ZR can improve root growth and enhance plants’ nutrient uptake capacity (Wittenmayer et al., 2012). In the leaves, the relative growth rates of ZR decreased during T1 and T2. While ZR synthesis generally has a positive impact on plant height (Gholami, 2018), our study indicates that uniconazole application inhibits OJ plant height, particularly at high doses. Thus, the reduction in OJ plant height correlates with decreased ZR synthesis during T1 and T2.

In terms of Abscisic Acid (ABA), unlike Gibberellic acid 3 (GA_3_) and Indole-3-Acetic Acid (IAA), the relative growth rates of ABA in various parts of *Ophiopogon japonicus* (OJ) increased during T2, indicating that uniconazole treatment influences ABA synthesis in these areas. T2 occurs during the winter’s low-temperature period (mid-December to late January), and the increase in ABA synthesis may help OJ withstand cold conditions, enhancing its frost resistance. Similar findings have been reported in studies on rapeseed and tomatoes (Smoleńska-Sym et al., 1995; Finkelstein and Rock, 2002), where cold environments trigger ABA synthesis to help plants cope with adverse conditions. This period also marks a critical time for the accumulation of materials in OJ root tubers, particularly carbohydrates. Our research shows that uniconazole application significantly increases the total polysaccharide content in Ophiopogonis Radix, likely due to enhanced ABA synthesis. Previous studies have indicated that ABA promotes the accumulation of anthocyanins and sucrose (Saito et al., 2022). During T1, only OJ rhizome and nutritive root showed an increase in ABA growth rates. This period corresponds with the first peak in root tuber swelling, and ABA may influence the transport of cytokinins, affecting root elongation and nutrient uptake. The increased ABA synthesis in OJ rhizome and nutritive root may relate to root tuber swelling and material accumulation, with similar conclusions drawn from studies on Arabidopsis, corn, and barley (Roberts and Snowman, 2000; Carrió-Seguí et al., 2016; Vysotskaya et al., 2021). Plant leaves are the primary sites for rapid ABA biosynthesis in response to external stressors. The upregulation of the NCED1 gene involved in ABA biosynthesis is more pronounced in leaves compared to other plant tissues, underscoring their central role in ABA production (Zhang et al., 2018). During the swelling phase, OJ leaves undergo environmental temperature fluctuations, which suggests a need for further research to optimize ABA application in agricultural practices, as this is vital for enhancing OJ production.

### 4.3 Uniconazole remodels morphology-quality equilibrium in Ophiopogonis Radix

The *Ophiopogon japonicus* (OJ) root tubers enlarge due to various genetic and environmental factors (Boyer, 2009; Sun et al., 2020). In untreated plants, the swelling of OJ root tubers typically occurs at the end nearest the root tip, resulting in a spindle-like shape. However, with Uniconazole treatment, the swelling shifts to the end closer to the rhizome, or occurs immediately upon formation, leading to shorter storage roots. This results in a cylindrical or dumbbell shape for the root tubers, which become thicker and larger in volume. These changes in swelling location and morphology are related to Uniconazole’s effects, such as promoting root growth (Feng et al., 2020) and increasing root tip numbers (Zhou et al., 2021); higher doses lead to more pronounced changes. The morphological alterations of the OJ root tubers significantly affect the quality of the processed medicinal material, Ophiopogonis Radix (OR). This is evidenced by substantial changes in key quality indicators, including the length, diameter, and dry weight of the single OR. Our study indicates that uniconazole treatment enhances both the length and diameter of the OR; the effects are more pronounced at higher doses, with maximum increases of 21.75% in length and 12.36% in diameter. These changes subsequently lead to a significant increase in single OR weight (Dry weight), with a maximum increase of 49.53%. The alterations in these three quality indicators surpass the standards set by the Chinese Pharmacopoeia (Commission C P, 2020) for Ophiopogonis Radix, thereby conflicting with the established quality requirements. In addition to affecting external characteristics, uniconazole treatment also significantly influences the intrinsic quality of Ophiopogonis Radix, particularly its core active ingredient, saponins. While Uniconazole treatment does lead to increases in extract (by 0.76% to 2.32%), total polysaccharide content (by 9.13% to 17.25%), total flavonoid content (by 9.95% to 23.28%), and specific saponin compounds (Methylophiopogonanone A by 27.81% to 56.95% and Methylophiopogonanone B by 11.01% to 30.94%), it also results in a marked decrease in the core efficacy indicators, specifically total saponin content and Ophiopogonin D. The total saponin content can decrease by as much as 35.90%, while Ophiopogonin D can decrease by up to 63.94%.

Ophiopogonin D is a key marker compound and representative bioactive ingredient in the quality assessment of Ophiopogonis Radix (OR). It plays a dominant role in defining the medicinal value of the herb and is closely associated with the traditional therapeutic functions attributed to *Ophiopogon japonicus*. Pharmacological studies have confirmed that Ophiopogonin D offers protective effects against skeletal and cardiovascular diseases, exhibits anti-aging properties, and improves cognitive deficits (Chen et al., 2024). It has also been shown to alleviate oxidative stress and inflammation in diabetic nephropathy, thereby improving kidney function (Qiao et al., 2020); regulate inflammatory responses in conditions such as colitis and acute lung injury (Wang et al., 2022a; Shen et al., 2024); and mitigate metabolic syndrome in mice induced by a high-fat diet, helping to control obesity, hyperglycemia, insulin resistance, and fatty liver disease (Chen et al., 2018). These diverse pharmacological activities strongly align with the recognized therapeutic actions of OR, such as blood sugar reduction, cardiovascular protection, immune enhancement, anti-inflammatory, and anti-tumor effects. This multidimensional evidence reinforces the role of Ophiopogonin D as a representative compound that largely defines the medicinal quality of OR. However, foliar application of uniconazole has been shown to alter the morphological characteristics of OR and significantly reduce the levels of Ophiopogonin D, thereby compromising its quality and potentially diminishing its clinical efficacy. Future studies should focus on linking uniconazole treatment variables with metabolic pathway changes to unravel the molecular mechanisms underlying these alterations in key compounds. Such efforts will support the development of more precise and dynamic quality standards for Ophiopogonis Radix, ensuring both its safety and therapeutic effectiveness in clinical applications.

Due to the perceived benefits of Uniconazole on OR yield, some local farmers have begun to use it as a fertilizer, believing that higher application rates yield better results. This practice creates a conflict between production and theory; while Uniconazole may increase yields, it does not guarantee the efficacy of Ophiopogonis Radix as a medicinal product, thereby raising concerns about safety and human health. The current levels of agricultural productivity make it challenging to resolve this issue, leading to a situation where the scientific, rational, and safe use of growth regulators and pesticides is merely theoretical. Achieving large-scale, standardized production of medicinal herbs is essential to fundamentally address the many challenges faced in traditional medicine agriculture. By the time Ophiopogonis Radix is harvested, the uniconazole residue in plants treated with high doses (U3) is well below current maximum residue limits. The half-life of Uniconazole in Ophiopogonis Radix is approximately 19.69 days. This half-life varies in different crops; for instance, it is between 3.8 to 4.4 days in wheat (Zhang et al., 2013), 43.32 to 58.24 days in brown rice (Meng et al., 2018), and 2.8 days in strawberries (Saber et al., 2020). Understanding the half-life of Uniconazole is crucial for determining safe pre-harvest intervals and establishing maximum residue limits (MRL). Thus, the determination of Uniconazole’s half-life in Ophiopogonis Radix provides a basis for establishing the maximum residue limit (MRL) for this compound in the herb. Similarly, the half-life of Uniconazole in soil is approximately 19.75 days. Various factors, including soil type (such as clay content and organic matter) (Rao and Murty, 1980) and microbial activity (Liu et al., 2010), can significantly influence the degradation rate of Uniconazole in soil. Following the application of 131.20, degradation reached 99%. By the time of harvesting *Ophiopogon japonicus*, which occurs 150 days after spraying, Uniconazole degradation in soil can exceed 99%, ensuring that it does not adversely affect subsequent crops.

### 4.4 Regulatory gap in uniconazole residues for medicinal plants: imperative need for (maximum residue limits) MRL establishment

**Figure 11:**
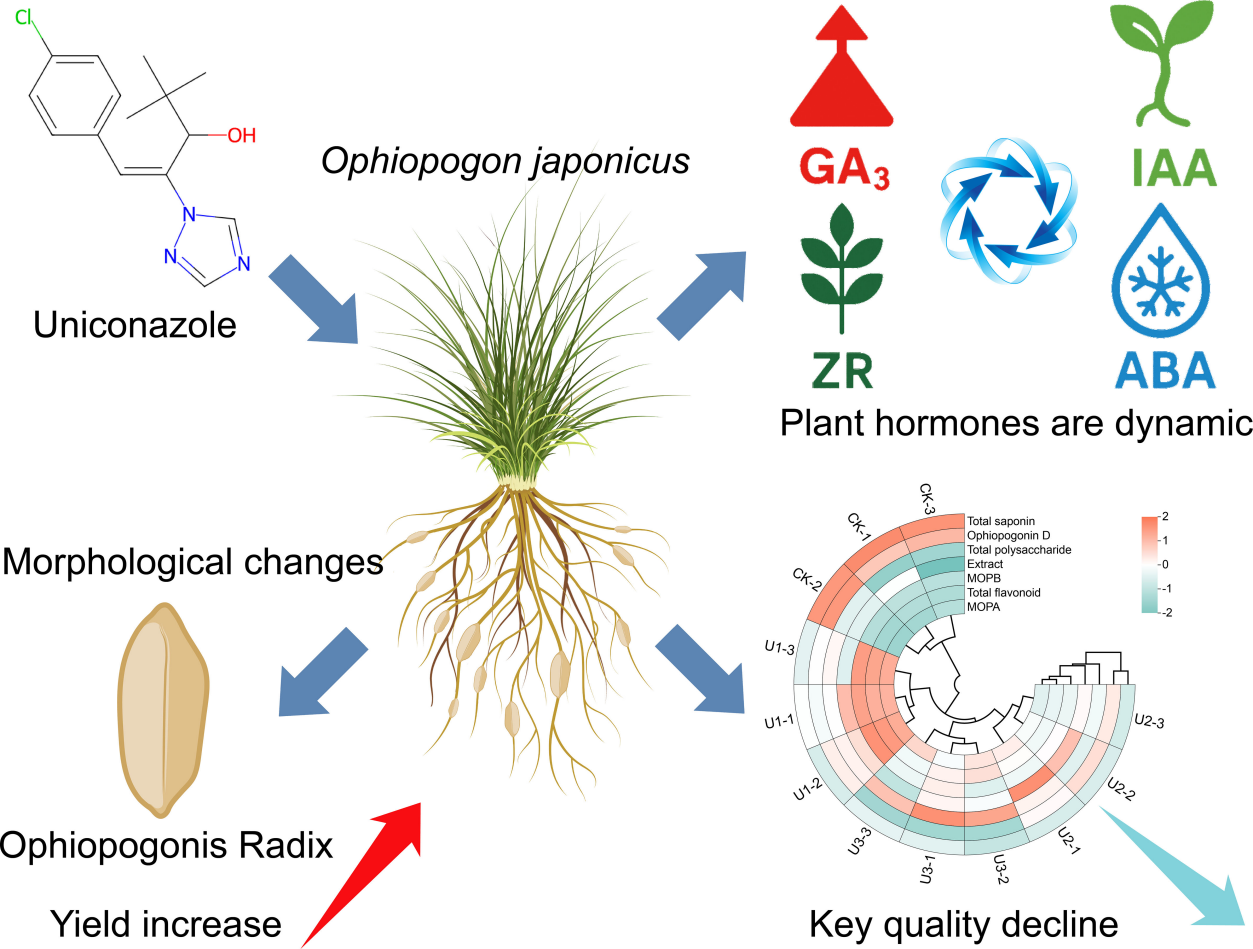
Overview diagram of the dual-effect balance and visualization analysis of uniconazole-mediated coordination between yield and quality in *Ophiopogon japonicus*.

Currently, several countries have strict regulations regarding the maximum residue levels of uniconazole. For example, Japan sets an MRL of 0.05 mg/kg for lettuce and onions and 0.1 mg/kg for rice, strawberries, cabbage, and beetroot. In China, Uniconazole has been widely used in agricultural production since its market introduction and is registered for use on various crops, including rice, wheat, peanuts, potatoes, rapeseed, tobacco, cotton, and fruit trees. According to the National Food Safety Standard for Maximum Residue Limits for Pesticides in Food (GB 2763-2016) (Ministry of Agriculture of China et al., 2016), there are established limits for Uniconazole residues in several crops, such as 0.05 mg/kg in soybeans and mangoes, and 0.2 mg/kg in rapeseed. However, there are currently few related limits in China, especially concerning the MRL for traditional Chinese medicine herbs like Ophiopogonis Radix, which raises concerns about product safety and market regulation. Therefore, it is imperative to define the maximum residue limits for Uniconazole in medicinal herbs, particularly considering its potential impact on food safety and human health (El-Makawy et al., 2006). The establishment of relevant limits for plant growth regulators is urgently needed to guide farmers in compliant practices, ensuring the safety of food and medicinal products for consumers. The visualized analysis of uniconazole-modulated dual-effect balance for synergistic yield-quality optimization in *Ophiopogon japonicus* is shown in **Figure 11**.

## 5 Conclusions

This study shows that foliar application of uniconazole alters the hormone balance in *Ophiopogon japonicus*, changing their shape and increasing yield by up to 101.59%. However, these changes reduce key medicinal compounds—total saponins and Ophiopogonin D—by as much as 35.90% and 63.94%, which may compromise clinical effectiveness and increase market adulteration risks. Although uniconazole residues are low at harvest, the trade-off between yield and quality is clear.

To address this issue, we propose a comprehensive strategy. First, it is essential to deepen our understanding of hormone–metabolite interactions and develop precision application techniques that balance yield and quality. This includes establishing dose–response models for plant hormones and monitoring dynamic changes in bioactive metabolites. Second, existing quality standards should be updated to reflect current challenges. Residue accumulation must be assessed through phased or seasonal field trials, integrating both chemical residue testing and bioactivity evaluation. A regulatory framework should be established to ensure both application safety and pharmacological efficacy, alongside an environmental sustainability system—particularly focusing on the impact of uniconazole on soil microbial communities and non-target species diversity. Ultimately, improving the production of medicinal crops must prioritize product quality and safety. A crop-specific risk–benefit assessment model for Ophiopogonis Radix should be developed to align cultivation standards with clinical needs, thereby supporting sustainable, high-quality production practices.

## Data Availability

The raw data supporting the conclusions of this article will be made available by the authors, without undue reservation.
