# Supplementary material for: Uniconazole-mediated growth regulation in Ophiopogon japonicus: yield maximization vs. medicinal quality trade-offs
Source: Front Plant Sci. 2025 Jul 15;16:1542539. doi: 10.3389/fpls.2025.1542539 (PMC12308313; doi:10.3389/fpls.2025.1542539)
Supplement: Supplementary file 1 [file Table1.docx]

Table S1. Shows the hormone level changes in different parts of *Ophiopogon japonicus* during various tuber expansion stages.

| Types of Hormones | Parts | Treatment code | T1 | T2 | T3 |
| --- | --- | --- | --- | --- | --- |
| GA | leaves | CK | -1.83% | 0.60% | 0.32% |
|  |  | U3 | 5.71% | -0.77% | 0.97% |
|  | rhizomes | CK | -0.99% | 1.28% | -0.13% |
|  |  | U3 | -3.53% | -0.54% | 0.83% |
|  | nutritive roots | CK | 2.58% | 0.22% | 0.27% |
|  |  | U3 | 1.04% | -1.00% | -0.24% |
|  | storage roots | CK | -0.48% | 1.31% | -0.60% |
|  |  | U3 | 2.48% | -0.80% | 0.18% |
|  | root tubers | CK | -1.28% | 1.49% | 0.13% |
|  |  | U3 | 4.72% | -1.25% | 1.12% |
| IAA | leaves | CK | 1.11% | 0.59% | 0.48% |
|  |  | U3 | 0.86% | -1.16% | 0.55% |
|  | rhizomes | CK | 1.58% | 0.95% | -0.23% |
|  |  | U3 | -0.92% | 0.47% | -0.73% |
|  | nutritive roots | CK | 6.68% | -0.32% | 0.47% |
|  |  | U3 | 4.67% | -1.12% | 0.17% |
|  | storage roots | CK | 0.93% | 0.63% | 0.23% |
|  |  | U3 | 1.41% | 0.33% | -0.98% |
|  | root tubers | CK | -0.57% | 0.71% | 0.52% |
|  |  | U3 | -1.13% | -0.09% | -0.18% |
| ZR | leaves | CK | 1.69% | 0.49% | 0.54% |
|  |  | U3 | 0.76% | -0.59% | 0.80% |
|  | rhizomes | CK | 0.79% | -0.17% | 1.41% |
|  |  | U3 | 5.62% | 0.00% | -0.65% |
|  | nutritive roots | CK | 1.58% | -0.36% | 0.49% |
|  |  | U3 | -0.23% | 0.05% | -0.01% |
|  | storage roots | CK | 1.59% | 0.44% | -0.27% |
|  |  | U3 | 3.34% | -0.81% | 0.98% |
|  | root tubers | CK | -0.06% | 1.13% | 0.62% |
|  |  | U3 | 3.99% | 0.13% | -1.21% |
| ABA | leaves | CK | 0.13% | -1.84% | 0.78% |
|  |  | U3 | -2.84% | -0.35% | 1.67% |
|  | rhizomes | CK | -0.18% | -1.02% | 0.15% |
|  |  | U3 | 1.44% | -0.35% | -0.32% |
|  | nutritive roots | CK | -2.50% | -1.03% | 0.60% |
|  |  | U3 | 3.39% | -0.08% | -0.90% |
|  | storage roots | CK | 1.45% | -0.53% | 0.06% |
|  |  | U3 | -1.77% | 0.93% | 0.26% |
|  | root tubers | CK | 0.18% | -1.43% | 0.55% |
|  |  | U3 | -3.62% | -0.80% | 0.36% |
